# Impact of the gut microbiota on the development of obesity and type 2 diabetes mellitus

**DOI:** 10.3389/fmicb.2014.00190

**Published:** 2014-04-29

**Authors:** Isabel Moreno-Indias, Fernando Cardona, Francisco J. Tinahones, María Isabel Queipo-Ortuño

**Affiliations:** ^1^Unidad de Gestion Clínica de Endocrinología y Nutrición, Laboratorio del Instituto de Investigación Biomédica de Málaga (IBIMA), Hospital Universitario de Málaga (Virgen de la Victoria)Málaga, Spain; ^2^Centro de Investigación Biomédica en Red de Fisiopatología de la Obesidad y la NutriciónMadrid, Spain

**Keywords:** gut microbiota, obesity, type 2 diabetes mellitus, inflammation, LPS, SCFA

## Abstract

Obesity and its associated disorders are a major public health concern. Although obesity has been mainly related with perturbations of the balance between food intake and energy expenditure, other factors must nevertheless be considered. Recent insight suggests that an altered composition and diversity of gut microbiota could play an important role in the development of metabolic disorders. This review discusses research aimed at understanding the role of gut microbiota in the pathogenesis of obesity and type 2 diabetes mellitus (TDM2). The establishment of gut microbiota is dependent on the type of birth. With effect from this point, gut microbiota remain quite stable, although changes take place between birth and adulthood due to external influences, such as diet, disease and environment. Understand these changes is important to predict diseases and develop therapies. A new theory suggests that gut microbiota contribute to the regulation of energy homeostasis, provoking the development of an impairment in energy homeostasis and causing metabolic diseases, such as insulin resistance or TDM2. The metabolic endotoxemia, modifications in the secretion of incretins and butyrate production might explain the influence of the microbiota in these diseases.

## INTRODUCTION

The prevalence of obesity and its associated disorders, such as type 2 diabetes mellitus (TDM2), has increased substantially worldwide over the last decades. Recent insight suggests that an altered composition and diversity of gut microbiota could play an important role in the development of metabolic disorders. Most of the gut microorganisms reside in the large intestine, which contains an estimated 10^11-12^ bacterial concentrations per gram of content (Leser and Molbak, 2009). These gut microbiota play a number of physiological roles involving digestion, metabolism, extraction of nutrients, synthesis of vitamins, prevention against colonization by pathogens, and immunomodulation (Jumpertz et al., 2011; Purchiaroni et al., 2013). In addition to an increased energy harvest from the diet, several mechanisms, including chronic low-grade endotoxemia, regulation of biologically active fatty acid tissue composition, and the modulation of gut-derived peptide secretion, have been proposed as links between gut microbiota and obesity (Musso et al., 2010). However, the contribution of gut microbiota to obesity and diabetes in humans is unclear. This is probably for various reasons, such as the fact that the heterogeneous etiology of obesity and diabetes can be associated with different microbes, studies have involved participants of diverse ethnic origin and food habits, the large inter-individual variation in the composition of gut microbiota, and in particular the different methods that have been used to profile the microbiota in these studies (Tremaroli et al., 2012). On the other hand, the differences between gut microbiota in lean and obese individuals as well as the impact of diet in the composition of the gut microbiome are still not wholly understood. Thus, manipulation of the gut microbiome represents a novel approach to treating obesity although it is in no way a substitute for diet and exercise. This review discusses the research conducted in understanding the role of gut microbiota in the pathogenesis of obesity and TDM2.

## GUT MICROBIOTA COMPOSITION

Microorganisms colonize all surfaces of the human body that are exposed to the environment, with most residing in the intestinal tract. Bacterial communities at a particular body site have more similarity among different subjects than in the same subject but at different body sites; i.e., there is more similarity between oral bacterial communities of different individuals than between the bacterial communities of the skin and the mouth in a single individual (Costello et al., 2009), although there is also considerable inter-individual variability (Costello et al., 2009; Robinson et al., 2010). The bacterial component of the microbiota has hardly been studied in recent years, driven by large-scale projects such as the Human Microbiome Project (Turnbaugh et al., 2007; Peterson et al., 2009) and MetaHIT (Qin et al., 2010). Research about gut microbiota, mainly using ribosomal 16S RNA and whole-genome sequencing (WGS – whole-genome shotgun; Turnbaugh et al., 2009b), has provided a general view of the commensal microbial communities and their functional capacity. For instance, in 2010, a catalog was established of 3.3 million gut microbial genes (Qin et al., 2010), with another wider catalog published soon after (Human Microbiome Project Consortium, 2012a,b). These studies have shown the great variability in microbiota composition among healthy subjects, even between twins sharing less than 50% of their bacterial taxons at the species level (Turnbaugh et al., 2010). However, this does not mean genetics has no role in the establishment and conformation of the gut microbiota, and it has been demonstrated that bacterial community composition is influenced by host-specific genomic locus (Benson et al., 2010; Koenig et al., 2011). Metagenomic studies have established that in spite of the high interpersonal variability, some bacterial groups share functionalities (Turnbaugh et al., 2009b; Burke et al., 2011). The main bacterial phyla are: Firmicutes (Gram-positive), Bacteroidetes (Gram-negative), and Actinobacteria (Gram-positive). Firmicutes is found in the highest proportion (60%), with more than 200 genera, the most important of which are: *Mycoplasma*, *Bacillus*, and *Clostridium*; Bacteroidetes and Actinobacteria each comprise about 10% of the gut microbiota, with the rest belonging to over 10 minority families. In total there are more than 1000 different species in the gut. It has also been suggested that the microbiota of most individuals can be categorized into three predominant enterotypes dominated by three different genera: *Bacteroides*, *Prevotella*, and *Ruminococcus*, which are independent of age, gender, ethnicity, or body mass index (BMI; Benson et al., 2010; Arumugam et al., 2011). Nevertheless an important debate has recently started about the concept of enterotypes (Jeffery et al., 2012; Yong, 2012), with a number of studies failing to identify the three distinct categories described by Arumugam et al., 2011 (Claesson et al., 2012; Huse et al., 2012).

## MICROBIOTA ESTABLISHMENT

Changes are produced in our microbiota from birth to adulthood. The fetal intestinal tract is sterile until birth, after which the newborn tract begins to be colonized. Infants are exposed to a great variety of microorganisms from different environments during and immediately after birth, either in their encounter with their maternal vagina or by the cutaneous microorganisms depending on the type of delivery (Adlerberth and Wold, 2009; Dominguez-Bello et al., 2010). Infants born vaginally have similar communities to those found in the vaginal microbiota of their mothers. In contrast, those born by Caesarian section have the characteristic microbiota of the skin, with taxons like *Staphylococcus* and *Propionibacterium* spp. (Dominguez-Bello et al., 2010). Moreover, these infants have lower intestinal bacteria counts with less diversity in the early weeks of life (Grölund et al., 1999; Axad et al., 2013). Another factor influencing the microbiota concerns the method of feeding. The microbiota of breast-fed infants is dominated by *Bifidobacterium* (Turroni et al., 2012; Yatsunenko et al., 2012) and *Ruminococcus* (Morelli, 2008), with significantly lower rates of colonization by *Escherichia coli*, *C. difficile*, *Bacteroides fragilis*, and *Lactobacillus* than those observed in exclusively formula-fed infants (Penders et al., 2006). The microbiota of formula-fed infants is more complex and includes enterobacterial genera, *Streptococcus*, *Bacteroides*, and *Clostridium*, as well as *Bifidobacterium* and *Atopobium* (Bezirtzoglou et al., 2011). But, the composition of the microbiota changes with the introduction of solid foods and a more complex and stable community similar to the adult microbiota becomes established at 2–3 years of age (Palmer et al., 2007; Koenig et al., 2011; Ravel et al., 2011; Yatsunenko et al., 2012), with Firmicutes and Bacteroidetes predominating. During adulthood the microbiota is relatively stable until old age, when this stability is reduced (McCartney et al., 1996). The ELDERMET consortium studied the microbiota of elderly Irish subjects, finding a different characteristic microbiota composition to that of young persons, particularly in the proportions of *Bacteroides* spp. and *Clostridium* groups (Claesson et al., 2011).

## EFFECT OF DIET ON THE TEMPORAL DYNAMICS OF MICROBIOTA

Human-related microorganisms have been enumerated and categorized (Costello et al., 2009) and their temporal dynamics have been described (Caporaso et al., 2011). To understand the stability of microbiota within an individual over time is an important step to predict diseases and develop therapies to correct dysbiosis (microbial community mismatches). Data from longitudinal studies show the microbiota composition is relatively stable in healthy adults over time and is only transiently altered by external disturbances such as diet, disease, and environment (Delgado et al., 2006). Particularly, changes in diet have shown important effects on the composition of the intestinal microbiota. Indeed, dietary changes could explain 57% of the total structural variation in gut microbiota whereas changes in genetics accounted for no more than 12% (Zhang et al., 2010). Diet provides nutrients for both the host and the bacteria of the gastrointestinal tract. Changes in the composition of the gut microbiota in response to dietary intake take place because different bacterial species are better equipped genetically to utilize different substrates (Scott et al., 2008). Many studies have demonstrated that an increase in fat intake produces an increase in the Gram-negative/Gram-positive index of our microbiota. Recent studies have found that mice [humanized germ-free (GF)] changed from a diet low in fat and rich in vegetable polysaccharides to a diet rich in fat and sugar and low in plant polysaccharides (western diet) changed their microbiota in just 1 day. Mice on the “western diet” experienced an increase in the abundance of bacteria of the phylum Firmicutes and a decrease in the abundance of those of the phylum Bacteroidetes (Turnbaugh et al., 2009a,b). Hildebrandt et al. (2009) also found important changes in the abundance of the gut microbiota of mice after changing from a standard chow to a high-fat diet, which was associated with a decrease in the abundance of bacteria of the phylum Bacteroidetes and an increase in that of both Firmicutes and Proteobacteria phyla. Moreover, murine studies have shown that carbohydrate-reduced diets result in enriched populations of bacteria from the Bacteroidetes phyla (Walker et al., 2011) while calorie-restricted diets prevent the growth of *C. coccoides*, *Lactobacillus* spp., and *Bifidobacteria* spp., which are all major butyrate producers required for colonocyte homeostasis (Santacruz et al., 2009). Only a limited number of human clinical trials have assessed the effects of changes in dietary patterns on the intestinal microbiota (De Palma et al., 2009; Muegge et al., 2011; Walker et al., 2011). In a controlled-feeding study with humans consuming a high-fat/low-fiber or low-fat/high-fiber diet, notable changes were found in gut microbiota in just 24 h, highlighting the rapid effect that diet can have on the intestinal microbiota (Wu et al., 2011). Interestingly, De Filippo et al. (2010) found that European children have a microbiota depleted of Bacteroidetes and enriched in Enterobacteriaceae compared to rural African children, which the authors attributed to low dietary fiber intake by Europeans (Wu et al., 2011). These authors postulated that gut microbiota co-evolved with the plant-rich diet of the African children, allowing them to maximize energy extraction from dietary fiber while also protecting them from inflammation and non-infectious intestinal diseases (De Filippo et al., 2010). Another study demonstrated that subjects consuming a vegan or vegetarian diet had a lower stool pH and significantly lower total counts of culturable *Bacteroides* spp., *Bifidobacterium* spp., *E. coli*, and Enterobacteriaceae spp. than controls (Zimmer et al., 2011). A vegetarian diet has also been shown to decrease the amount and change the diversity of *Clostridium* cluster IV and *Clostridium* clusters XIV and XVII (Liszt et al., 2009). However, large well-controlled trials are needed to elucidate the mechanisms that link dietary changes to alterations in microbial composition as well as the implications of key population changes for health and disease.

## MODULATION OF GUT MICROBIOTA DIVERSITY BY ANTIBIOTICS

Much evidence now exists concerning an important change in our microbiota over recent decades, with some species increasing and others decreasing, though one of the most striking findings is that in developed countries there is a loss in the diversity of our microbiota. One of the most important factors that can disturb microbiota composition is the increased use of antibiotic treatment. There is evidence of important alterations in microbiota after antibiotic treatment (Sullivan et al., 2001; Jernberg et al., 2007; Dethlefsen et al., 2008). Although affected taxons vary among subjects, some taxons are not recovered even several months after treatment, and in general, there is a long-term reduction in bacterial diversity after the use of antibiotics (Jernberg et al., 2010; Dethlefsen and Relman, 2011). A correlation has recently been proposed between the increasing global use of antibiotics and weight gain or obesity in humans (Thuny et al., 2010). Several studies have indicated that some antibiotics are associated with weight gain in malnourished children, neonates, and adults (Ajslev et al., 2011; Trehan et al., 2013), but the precise mechanisms by which antibiotics improve weight are not well characterized. It has been suggested that antibiotics, such as avoparcin (a glycopeptide structurally related to vancomycin), exert selective pressure on Gram-positive bacteria and that gut colonization by *Lactobacillus* spp., which are known to be resistant to glycopeptides, used as a growth promoter in animals and found at a high concentration in the feces of obese patients, could be responsible for the weight gain observed in patients who had been treated with vancomycin. These data suggest that nutritional programs and follow-up of weight should be undertaken in patients under such treatment (Thuny et al., 2010). Other recent studies have also demonstrated the beneficial effects of antibiotics on metabolic abnormalities in obese mice, giving rise to reduced glucose intolerance, body weight gain, metabolic endotoxemia, and markers of inflammation and oxidative stress (Bech-Nielsen et al., 2012). Moreover, these effects were associated with a reduced diversity of gut microbiota (Murphy et al., 2013). Antibiotic treatment combined with a protective hydrolyzed casein diet has been found to decrease the incidence and delay the onset of diabetes in a rat model (Brugman et al., 2006). A recent study also reported that antibiotic-treated humans showed greater and less balanced sugar anabolic capabilities than non-treated individuals (Hernandez et al., 2013). However, the majority of clinical studies are focused primarily on the characterization of the composition and diversity of gut microbes, it remaining uncertain whether antibiotic-induced gut microbiota alteration in human subjects with metabolic disorders is associated with improvements in metabolic derangements as observed in animal studies.

## ROLE OF GUT MICROBIOTA IN METABOLIC DISEASES

Recent decades have seen an increase in the prevalence of metabolic diseases in developed countries. Environmental factors, such as the increase in energy intake and the decrease in physical activity, have been considered causes of this spectacular increase in the prevalence of metabolic diseases. However, even when the energy intake does not increase and physical activity does not decrease, the prevalence continues growing exponentially, so other environmental factors must be taken into account, including changes in gut microbiota. One of the challenges is to elucidate the molecular origin of metabolic diseases, though the great diversity and social differences among humans make this difficult. During the last half century, with the advances in molecular biology, researchers have been investigating the genetics of metabolic diseases. In spite of the great efforts and the identification of some mutations in the genome, no global view has yet been established. The discovery of candidate genes in studies of pangenomic associations (GWAS – genome-wide association studies) has helped to identify new genes associated with sensitivity/resistance to diabetes and extreme metabolic phenotypes (Jacquemont et al., 2011). However, the global diversity of metabolic diseases cannot be explained, especially given the studies in monozygotic twins, discordant for TDM2 and obesity (Medici et al., 1999; Beck-Nielsen et al., 2003).

A second step toward the comprehension of the origin of metabolic diseases involves epigenetic and environmental factors. A drastic change in feeding habits in which dietary fiber has been replaced by a high fat diet contributes to the origin of metabolic diseases. However, this simple concept cannot explain why some people are sensitive and others are resistant to the development of these metabolic diseases. In mice, a metabolic adaptation is frequently observed (Burcelin et al., 2002). Genetically identical mice in the same box and with a fat-rich diet for 6–9 months can develop both obesity and diabetes, or only one of the diseases. There is a need to find a new paradigm that takes into account the genetic diversity, the environmental factor impact, the rapid development of metabolic diseases, and the individual behavior to develop diabetes and obesity. The conclusion reached concerns the concept of personalized medicine in which the individual characteristics should be identified in order to adapt a suitable therapeutic strategy for small patient groups.

## INFLUENCE OF GUT MICROBIOTA COMPOSITION IN THE DEVELOPMENT OF OBESITY

Studies during the last decade have associated the gut microbiota with the development of metabolic disorders, especially diabetes and obesity. Although incompletely understood, the gut microbiota is implicated in the programing and control of many physiological functions, including gut epithelial development, blood circulation, innate and adaptative mechanisms (Mackie et al., 1999; Dethlefsen et al., 2006). A new theory shows microbiota as a contributor to the regulation of energy homeostasis. Thus, with the environmental vulnerabilities, gut microbiota could provoke the development of impairment in energy homeostasis, causing metabolic diseases.

The first discovery was related to the fact that mice with a mutation in the leptin gene (metabolically obese mice) have different microbiota as compared with other mice without the mutation (Ley et al., 2005). In this obese animal model, the proportion of the dominant gut phyla, Bacteroidetes and Firmicutes, is modified with a significant reduction in Bacteroidetes and a corresponding increase in Firmicutes (Ley, 2010). Ley et al. (2006) were the first to report an altered gut microbiota similar to that found in obese mice (a larger proportion of Firmicutes and relatively fewer Bacteroidetes) in 12 obese subjects compared with 2 lean controls. Later, Armougom et al. (2009) confirmed a reduction in Bacteroidetes accompanied by a rise in *Lactobacillus* species belonging to the Firmicutes phylum. Turnbaugh et al. (2009b) and Furet et al. (2010) showed a different pattern based on a lower representation of Bacteroidetes (*Bacteroides/Prevotella*) in obese individuals with no differences in Firmicutes phylum. Collado et al. (2008) reported increases in species belonging to both Firmicutes (*Staphylococcus aureus*) and Bacteroidetes (*Bacteroides/Prevotella*) in overweight women. Million et al. (2012) described changes in the composition of Firmicutes based on an increase in *Lactobacillus reuteri* coupled with a reduction in *L. paracasei* and *L. plantarum*. Finally, other studies have found no differences between Firmicutes and Bacteroidetes at the phylum level (Duncan et al., 2008; Mai et al., 2009; Jumpertz et al., 2011).

**FIGURE 1:**
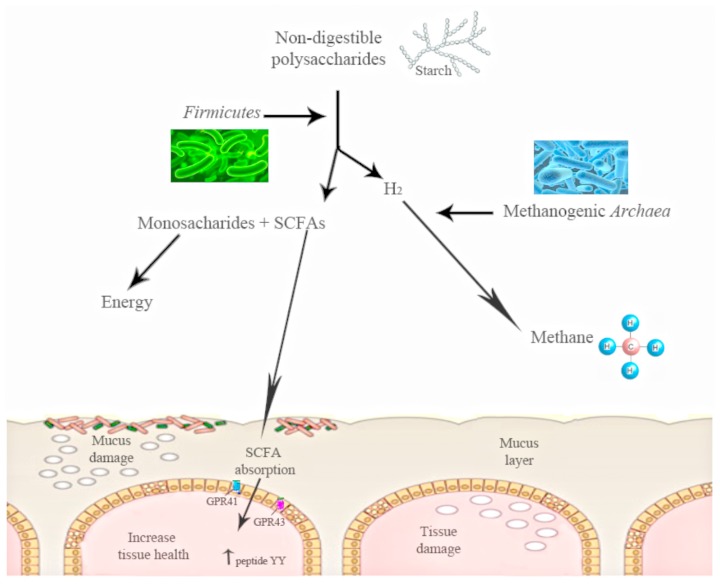
**The action of gut microbiota is needed to digest some polysaccharides.** Gut microbiota converts polysaccharides into monosac-charides and short-chain fatty acids (SCFA). These SCFAs are able to bind and activate two G-protein-coupled receptors (GPR41 and GPR43) of the gut epithelial cells. The activation of these receptors induces peptide YY. Starch digestion is an example of this process: H_2_is produced, and its increase inhibits starch digestion, moment at which other bacterial groups work and transform the H_2_ into methane.

The shift in the relative abundance observed in these phyla is associated with the increased capacity to harvest energy from food and with increased low-grade inflammation. The increase in Firmicutes and the decrease in the proportion of Bacteroidetes observed in obese mice could be related with the presence of genes encoding enzymes that break down polysaccharides that cannot be digested by the host, increasing the production of monosaccharides and short-chain fatty acids (SCFA) and the conversion of these SCFA to triglycerides in the liver (**Figure 1**). These SCFAs are able to bind and activate two G-protein-coupled receptors (GPR41 and GPR43) of the gut epithelial cells. The activation of these receptors induces peptide YY secretion, which suppresses gut motility and retards intestinal transit. By this mechanism of SCFA-linked G-protein-coupled receptor activation, the gut microbiota may contribute markedly to increased nutrient uptake and deposition, contributing to the development of metabolic disorders (Erejuwa et al., 2014). Moreover, gut microbiota have also been shown to decrease the production of the fasting-induced adipose factor [FIAF; a secreted lipoprotein lipase (LPL)] by the intestinal cells, which inhibits LPL activity, increasing the storage of liver-derived triglycerides (Backhed et al., 2007).

Turnbaugh et al. (2006), in a study using ob/ob mice, found a reduced calorie content in the feces of obese mice as compared with lean mice. Other studies have suggested that obese subjects might be able to extract more energy from nutrients due to hydrogen transfer between taxa. In fact, a simultaneous increase in both hydrogen-producing Prevotellaceae and hydrogen-utilizing methanogenic Archaea has been previously associated with obesity by Zhang et al. (2009), suggesting a higher energy harvest in obese patients. For instance, intestinal starch digestion produces hydrogen, the increase of which inhibits digestion and methanogenic Archaea are able to transform this hydrogen into methane (**Figure 1**). Thus, there is a specific microbiota that obtains more energy from the same energy intake (Turnbaugh et al., 2009a). These findings agree with the observation in which GF mice fed with a fat-rich diet gained less weight than conventional mice (Backhed et al., 2004).

The most relevant experiment dealing with the causality between microbiota and obesity was done by Turnbaugh et al. (2006). In this study, they demonstrated that microbiota transplantation from genetically obese mice to axenic mice provokes a very significant weight increase compared with the axenic mice transplanted with the microbiota from lean mice.

Surprisingly, the phenotype with increase capacity for energy harvest is simply transmitted by transplantation of the obesity-associated gut microbiota in to healthy and lean donors (Turnbaugh et al., 2006, 2008). But within a phylum, not all the genera have the same role, so that bacterial genera have been related with either beneficial or harmful characteristics associated within the same phylum. Kalliomäki et al. (2008) undertook a prospective study in which they followed 49 children from birth to 7 years of age. Stool was collected at 6 and 12 months of life and it was found that the children who were 7 years old with a normal weight had a higher number of *Bifidobacterium* spp. and a smaller number of *Staphylococcus aureus* than the children who became overweight several years later. The authors concluded that the alteration in the microbiota precedes the alteration in weight, an explanation that is relevant for obesity prevention. The authors also proposed that *Staphylococcus aureus* may act as a trigger of low-grade inflammation, contributing to the development of obesity (Kalliomäki et al., 2008).

On the other hand, *Lactobacillus* spp. and bifidobacteria represent a major bacterial population of the small intestine where lipids and simple carbohydrates are absorbed, especially in the duodenum and jejunum. Recent publications reveal that the *Bifidobacteria* and *Lactobacillus* are not all the same and they may have different characteristics according to the species. For example, within the genus *Lactobacillus*, *L. plantarum*, and *L. paracasei* have been associated with leanness whereas *L. reuteri* is associated with obesity (Million et al., 2012). Moreover, Drissi et al. (2014) have shown that weight gain-associated *Lactobacillus* spp. encode more bacteriocins and appear to lack enzymes involved in the catabolism of fructose, a defense against oxidative stress and the synthesis of dextrin, L-rhamnose and acetate than weight protection-associated *Lactobacillus* spp., which encodes for a significant gene amount of glucose permease. Regarding lipid metabolism, thiolases were only encoded in the genome of weight gain-associated *Lactobacillus* spp. The results of this study revealed that weight protection-associated *Lactobacillus* spp. have developed defense mechanisms for enhanced glycolysis and defense against oxidative stress while weight gain-associated *Lactobacillus* spp. possess a limited ability to break down fructose or glucose and might reduce ileal brake effects (Drissi et al., 2014).

## MICROBIOTA AND ITS RELATIONSHIP WITH TYPE 2 DIABETES MELLITUS

Type 2 diabetes mellitus is the consequence of an increase in the production of glucose in the liver and a deficit in the secretion and action of insulin. Other physiological functions are altered, such as the central and autonomous nervous systems, leading to an impaired secretion of hormones like glucagon and incretins. However, a common feature of obesity and TDM2 is the presence of a low-grade inflammatory component described in tissues involved in metabolism regulation, such as the liver, adipose tissue, and muscles (Pickup and Crook, 1998). This metabolic inflammation is characterized by a moderate excess in cytokine production, including interleukin (IL)-6, IL-1, or tumor necrosis factor alpha (TNF-α), that injures cellular insulin signals and contributes to insulin resistance and diabetes (Hotamisligil, 2006; Shoelson et al., 2006). Weight increase would be an initiating factor of low-grade inflammation. When adipocyte hypertrophy is produced as a response to excess energy intake, an increase in TNF-α production in the adipose tissue is also produced and this stimulates the production of chemotactic factors resulting in adipose tissue being infiltrated by proinflammatory macrophages that produce an increase in the production of IL-6 and IL-1. Recently, two studies have shown that the intestinal microbiome might be an important contributor to the development of TDM2. Both studies also showed that TDM2 subjects were characterized by a reduction in the number of Clostridiales bacteria (*Roseburia* species and *Faecalibacterium prausnitzii*), which produce the SCFA butyrate (Qin et al., 2012; Karlsson et al., 2013). Also, another study found microbiota changes in patients with diabetes or insulin resistance as compared with subjects without alterations in carbohydrate metabolism (Serino et al., 2013). In addition, changes in the amount of *Bifidobacterium*, *Lactobacillus*, and *Clostridium* as well as a reduced Firmicutes to Bacteroidetes ratio in gut microbiota have also been recently reported in type 1 diabetic children. This study also showed that bacteria involved in the maintenance of gut integrity were significantly lower in diabetic patients than in healthy controls (Murri et al., 2013). Similar changes in the composition of intestinal microbiota have also been reported in TDM2 patients (Larsen et al., 2010; Qin et al., 2012). Several other studies linking the gut microbiota to metabolic disorders, such as obesity, insulin resistance and diabetes mellitus, have been reviewed by other authors (Caricilli and Saad, 2013; Stachowicz and Kiersztan, 2013; Tagliabue and Elli, 2013). Moreover, probiotic (Amar et al., 2011) and prebiotic treatments (Cani et al., 2007b) control gut microbiota and metabolic diseases.

Various mechanisms have been proposed to explain the influence of the microbiota on insulin resistance and TDM2, such as metabolic endotoxemia, modifications in the secretion of the incretins and butyrate production.

**FIGURE 2:**
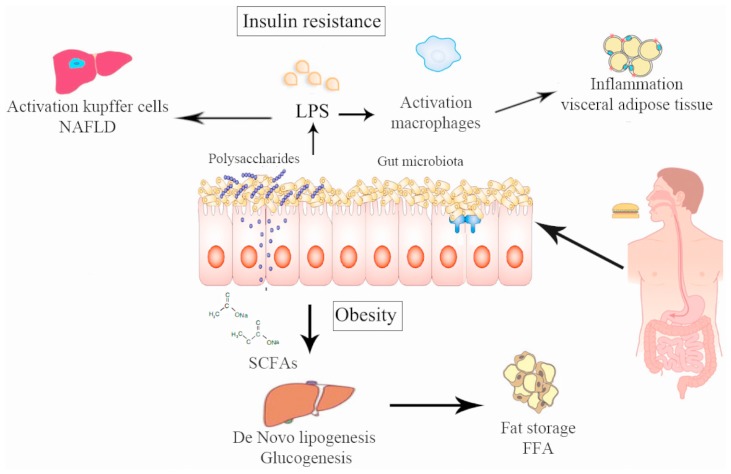
**Pathways via which intestinal microbiota can alter human metabolism producing obesity and insulin resistance.** (1) Chronic bacterial translocation due to increased intestinal permeability that can drive a systemic inflammation leading to macrophage influx into visceral adipose tissue, activation of hepatic Kupffer cells and insulin resistance. (2) Short-chain fatty acids normalize intestinal permeability and alter *de novo* lipogenesis and gluconeogenesis via reduction of free fatty acid production by visceral adipose tissue.

The lipopolysaccharides (LPS) are endotoxins commonly found in the outer membrane of Gram-negative bacteria that cause metabolic endotoxemia, which is characterized by the release of proinflammatory molecules (Manco et al., 2010). A rise in LPS levels has been observed in subjects who increased their fat intake (Amar et al., 2008). Similar results were found in mice (Cani et al., 2007b) and in mutant mice (like the leptin-deficient mice) even feeding with a normal diet (Cani et al., 2008), which suggests that a change in the proportion of Gram-negative bacteria in the gut or a change in the gut permeability were produced by the LPS rise in serum (Cani et al., 2008, 2009b) and this increase is directly related with the degree of insulin resistance. Cani et al. (2007a,b) reported that modulation of the intestinal microbiota by using prebiotics in obese mice acts favorably on the intestinal barrier, lowering the high-fat diet-induced LPS endotoxemia and systemic and liver inflammation (**Figure 2**). LPS are absorbed by enterocytes and they are conveyed into plasma coupled to chylomicrons (Clemente Postigo et al., 2012). In this way, dietary fats can be associated with increased absorption of LPS which in turn can be related with changes in the gut microbiota distinguished by a decrease in the *Eubacterium rectale*–*C. coccoides* group, Gram-negative *Bacteroides* and in *Bifidobacterium* (Caricilli and Saad, 2013). This causal role of LPS was demonstrated by infusing LPS in mice with a normal diet inducing hepatic insulin resistance, glucose intolerance, and an increase in the weight of adipose tissue (Cani et al., 2007a). It has been recently shown that the LPS-induced signaling cascade via Toll-like receptor 4 (TLR4) impairs pancreatic β-cell function via suppressed glucose-induced insulin secretion and decreased mRNA expression of pancreas-duodenum homebox-1 (PDX-1; Rodes et al., 2013). LPS binds to the CD14/TLR4 receptor present on macrophages and produces an increase in the production of proinflammatory molecules. When LPS injections were administrated to mice with a genetic absence of the CD14/TLR4 receptor they did not develop these metabolic characteristics and there was no start of TDM2 or obesity, showing the important role of LPS in the mechanism of CD14/TLR4. Moreover, knockout CD14/TLR4 mice were even more sensitive to insulin than wild type controls (Cani et al., 2007a; Poggi et al., 2007). LPS can also promote the expression of NF-κB (nuclear factor kappa-light-chain-enhancer of activated B cells) and activation of the MAPK (mitogen-activated protein kinase) pathway in adipocytes with several target genes (Chung et al., 2006).

An increase of *Bifidobacterium* spp. modulates inflammation in obese mice by an increase in the production of incretins like the glucagon-like peptide (GLP), also reducing intestinal permeability (Cani et al., 2009b). There is evidence that the rise in *Bifidobacterium* spp. produced by some prebiotics is accompanied by an increase in GLP1 and YY peptide secretions by the intestine. These two molecules have favorable effects, decreasing insulin resistance and the functionality of beta cells (Cani and Delzenne, 2009). In addition, modulation of the gut flora with prebiotics increases GLP2 production in the colon and this increase in GLP2 production is associated with higher expression of zonula occludens-1 (ZO-1), which improves the mucosal barrier function leading to a decrease in plasma LPS (Cani et al., 2009a,b). The study by Qin et al. (2012) showed that subjects with TDM2 suffered from a moderate intestinal dysbiosis and an increase in the number of various opportunistic gut pathogens, more than a change in a specific microbial species, having a direct association with the pathophysiology of TDM2. Specifically, they experienced a decrease in their butyrate-producing bacteria (Qin et al., 2012). This is significant because butyrate is the preferred source of energy, repair and maintaining cell health in the human digestive system. In the colon, the predominant butyrate-producing bacteria are the *C. coccoides* and the *Eubacterium rectale* groups. These changes in intestinal bacteria have recently been reported in patients with colorectal cancer (Wang et al., 2012) and in elderly people (Biagi et al., 2010). Thus, butyrate-producing bacteria could have a protecting role against a functional dysbiosis. Moreover, as other intestinal diseases show a loss of butyrate-producing bacteria with a commensurate increase in opportunistic pathogens, a possible hypothesis is that this change in the microbiota can cause an increase in susceptibility to a wide variety of diseases. The analysis of genetic bacterial functions shows an increase in functions related to the response to intestinal oxidative stress. This is of interest, because previous studies have shown that a high oxidative stress level is related to a predisposition to diabetic complications (Kashyap and Farrugia, 2011).

## CONCLUSION

Metabolic diseases are caused by many factors, including a higher consumption of energy-rich diets, reduced physical activity, and a hereditary disposition. In the past 6 years, much evidence suggests that gut microbiota may play an important role in the regulation of energy balance and weight in animal models and in humans. However, although metagenomic tools have provided an important amount of data concerning the characterization and the potential role of this gut microbiota in the development of human obesity and TDM2, the causal relationship between this microbiota and obesity still needs to be confirmed in humans. In the future, larger human studies conducted at the species level and taking into account all of the possible confounding variables (such as age, gender, ethnicity, diet, and genetic factors) are needed to allow us to use the gut microbiota composition and modulation as novel diagnostic or therapeutic strategies to treat obesity and TDM2.

## Conflict of Interest Statement

The authors declare that the research was conducted in the absence of any commercial or financial relationships that could be construed as a potential conflict of interest.
